# Breast Tumor Tissue Image Classification Using DIU-Net

**DOI:** 10.3390/s22249838

**Published:** 2022-12-14

**Authors:** Jiann-Shu Lee, Wen-Kai Wu

**Affiliations:** Department of Computer Science and Information Engineering, National University of Tainan, Tainan 700, Taiwan

**Keywords:** soft segmentation, DIU-Net, joint training

## Abstract

Inspired by the observation that pathologists pay more attention to the nuclei regions when analyzing pathological images, this study utilized soft segmentation to imitate the visual focus mechanism and proposed a new segmentation–classification joint model to achieve superior classification performance for breast cancer pathology images. Aiming at the characteristics of different sizes of nuclei in pathological images, this study developed a new segmentation network with excellent cross-scale description ability called DIU-Net. To enhance the generalization ability of the segmentation network, that is, to avoid the segmentation network from learning low-level features, we proposed the Complementary Color Conversion Scheme in the training phase. In addition, due to the disparity between the area of the nucleus and the background in the pathology image, there is an inherent data imbalance phenomenon, dice loss and focal loss were used to overcome this problem. In order to further strengthen the classification performance of the model, this study adopted a joint training scheme, so that the output of the classification network can not only be used to optimize the classification network itself, but also optimize the segmentation network. In addition, this model can also provide the pathologist model’s attention area, increasing the model’s interpretability. The classification performance verification of the proposed method was carried out with the BreaKHis dataset. Our method obtains binary/multi-class classification accuracy 97.24/93.75 and 98.19/94.43 for 200× and 400× images, outperforming existing methods.

## 1. Introduction

Cancer is currently one of the leading causes of human death worldwide, and for women, breast cancer is the second main cause of cancer death after lung cancer [1]. According to the International Agency for Research on Cancer (IARC), which is part of the World Health Organization (WHO) [2], the number of deaths caused by cancer is expected to increase to more than 27 million by 2030 [3]. Commonly used breast cancer clinical screening methods include mammography [4], breast ultrasound [5], biopsy [6] and other radiology imaging. Radiology images can help to identify abnormal areas. However, they cannot be used to determine whether the area is cancerous. The biopsy [6], where tissue is taken and studied under a microscope to see if cancer is present, is the only diagnostic procedure that can confirm whether a suspicious area is cancerous. After completing the biopsy, the diagnosis will be proceeded by the pathologists, who examine the tissue under a microscope, looking for cancerous cells. Pathologists determine cancerous regions and malignancy degree [7,8] by visually examining the regularities of cell shapes and tissue distributions. Microscopic examination of histological slides by a pathologist for diagnosis is considered the gold standard for making a definite diagnosis [9]. However, traditional manual diagnosis requires a lot of effort for a pathologist. Whether it is due to insufficient diagnosis experience or inattention, it is prone to make wrong diagnoses using the manual way. In contrast, the automatic classification of pathological images using computer-aided diagnosis (CAD) [10] can not only improve diagnostic efficiency but also provide more objective and stable diagnostic results.

In [11], a database of breast cancer histopathology images, called BreaKHis, was introduced by Spanhol et al. to overcome the problem of small data sets which is the main obstacle leading to the lack of development of a new analysis method. For texture features, popular textural descriptors were used, such as Local Binary Patterns (LBP) [12], Completed LBP (CLBP) [13], Local Phase Quantization (LPQ) [14], Grey-Level Co-occurrence Matrix (GLCM) [15], Threshold Adjacency Statistics [16], and Oriented fast and Rotated BRIEF (ORB) [17]. For classification, four different classifiers were used to assess the above feature sets including a 1-Nearest Neighbor (1-NN), Quadratic Discriminant Analysis (QDA), Support Vector Machines (SVM), and Random Forests. Chan et al. [18] calculated the fractal dimension of images of breast cancer slides and then classified them into benign or malignant images via SVM. Kahya et al. [19] presented an adaptive sparse support vector machine by combining the support vector machine with the weighted L1-norm to classify the breast cancer histopathology images. However, the performance of the above methods is limited due to the manual-based feature design methodology, which can be overcome by the convolutional neural network (CNN).

In the last decade, the CNN has achieved great success in image and video analysis and has received the attention of pathology image analysis researchers. Bardou et al. [20] compared two machine learning schemes for classifying breast cancer pathology images into benign and malignant. The first scheme is based on the extraction of a set of handcrafted features encoded by two coding models, bag of words and locality constrained linear coding, and trained by support vector machines. The second scheme is based on the design of CNNs. The experimental results show that CNNs outperformed the handcrafted feature-based classifier. Motlagh et al. [21] presented Inception and ResNet architectures to discriminate microscopic cancerous images. They demonstrated an automatic framework for breast tumor detection and classification of its subtypes. The above two methods employing the existing CNN models for feature extraction and classification cannot adapt to the innate characteristics of pathological images; hence, the classification performance is naturally limited. Jiang et al. [22], considering the characteristics of histopathological images, designed a new CNN architecture for the classification of breast cancer histopathology images using the small SE-ResNet module, which is named the breast cancer histopathology image classification network (BHCNet). This model was used for the automatic classification of breast cancer histology images into benign and malignant and eight subtypes. The study by Filipczuk et al. [23] confirms that nucleus features can effectively classify benign and malignant breast cancer. Based on this concept, George et al. [24] proposed a nucleus feature extraction method utilizing a convolutional neural network for automated breast cancer detection. Non-overlapping nuclei patches are detected from the images first, and then CNN is employed to extract features. A feature fusion approach with a support vector machine classifier is used to classify breast tumor images. However, since the detection of nuclei patches is obtained by traditional image processing methods, the accuracy is not high enough, and the patch detection errors or locating errors will affect the subsequent classification performance.

Complex structures of the pathological image and significant variations in the morphology of the same type of nucleus within and across images make pathological image classification a challenging task. The aforementioned BHCNet [22] can achieve decent classification results; however, there still exists room for further improvement. A feasible way to enhance breast tumor classification performance is to drive the CNN model to focus more on the nucleus regions in which the cancerous characteristics are contained. Based on this consideration, we proposed a segmentation–classification joint training mechanism to enhance the BHCNet. The segmentation module is responsible for learning the Nucleus Focus Map (NFM) in which the nucleus region corresponds to higher weights. The Nucleus Focus Weighted Image (NFWI) is obtained by multiplying the NFM by the input image, and then the NFWI is input to BHCNet. In response to the inconsistent size of nuclei in tissue slice images, we proposed a Dilated Inception U-Net (DIU-Net) model with better cross-scale description ability, which enhances the performance of nuclei segmentation. To adapt to the significant variations in the morphology of the same type of nucleus, this study proposed a Complementary Color Conversion Scheme (C3S) to enhance the generalization ability of the segmentation module. Since the area of nuclei in a pathological image is much smaller than the non-nucleus area, there exists a data imbalance phenomenon. This makes the trained segmentation module apt to pay more attention to the non-nucleus regions, resulting in segmentation bias. This study combined dice loss and focal loss to overcome this problem. The experimental results show that our method can indeed achieve better performance than the BHCNet [22]. In addition, our model has another advantage, that is, it can provide visual information of the model to the pathologist so that the pathologist can understand where the area concerned by the model is, and further enhance the pathologist’s trust in this model.

In summary, the main contributions of our study are as follows:

(1) A segmentation–classification joint training mechanism was proposed to enhance the classification performance for breast cancer pathology images.
(2) A Dilated Inception U-Net model with better cross-scale description ability was proposed to enhance the performance of nuclei segmentation.
(3) A Complementary Color Conversion Scheme was proposed to enhance the generalization ability of the nuclei segmentation.
(4) The proposed model can provide visual information of the model to the pathologist so that the pathologist can understand where the area concerned by the model is.

The rest of the paper is organized as follows: Section 2 is dedicated to the proposed segmentation–classification joint model and its training. In Section 3 and Section 4, the dataset, the implementation settings, performances and experimental results comparisons are given and discussed.

## 2. Method

### 2.1. System Architecture

**Figure 1:**
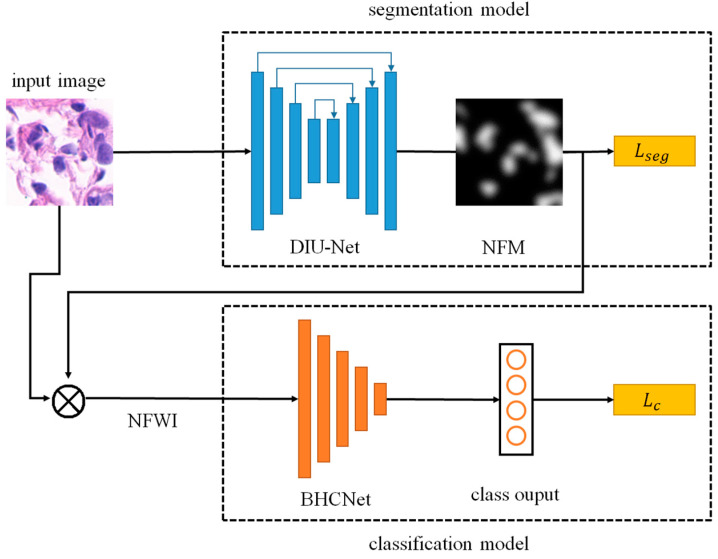
System architecture of the proposed method. For the binary classification case, the class output corresponds to benign/malignant, while for the multi-classification case, the class output corresponds to the eight subcategories.

The architecture of the proposed method is shown in Figure 1, including two parts: a segmentation network and a classification network. The architecture can be trained using the segmentation–classification joint training mechanism to achieve good performance for breast cancer classification. After the input image is softly segmented by the segmentation network, NFM is output and $L_{\mathrm{seg}}$ is the corresponding segmentation loss. The NFWI is obtained by multiplying the NFM by the input image, and then the NFWI is classified by the classification network and the corresponding classification loss $L_{c}$ is calculated. Detailed explanations of the different parts of the proposed network are provided in the following sections.

### 2.2. Segmentation Network

**Figure 2:**
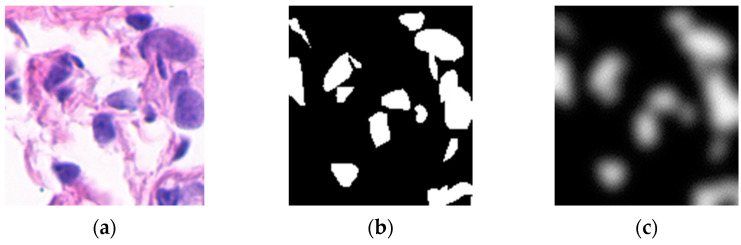
(**a**) A pathological tissue patch. (**b**) Hard segmentation result of (**a**). (**c**) Soft segmentation result of (**a**).

The nucleus features can be utilized to effectively classify benign and malignant breast cancer [23]. Pathologists pay more attention to the nucleus region when analyzing pathological images, so this study uses soft segmentation to imitate the visual focus mechanism of pathologists. Figure 2 shows the results of hard segmentation and soft segmentation for a pathological tissue patch exemplar in which the values of Figure 2c indicate the corresponding visual focus weights. To learn the corresponding NFM the segmentation network should possess pixel-level discrimination ability. U-net [25] was invented for semantic segmentation with an architecture that can be viewed as an encoder network followed by a decoder network. Unlike a classification network where the end result of the network is the only high-level semantic features, semantic segmentation not only requires discrimination at the pixel level but also a mechanism to project the discriminative features learned at different stages of the encoder onto the pixel space. U-Net is well recognized for its good performance in medical image segmentation tasks. To further enhance the segmentation performance of U-Net, some new architectures based on U-Net have been proposed in recent years. The most attractive one is the Attention U-Net [26]. Compared with R2U-Net [27] and U-Net++ [28], which need to use more parameters to improve performance, Attention U-Net can significantly improve performance with relatively fewer parameters. Therefore, this study uses Attention U-Net as the basic architecture of the segmentation network.

**Figure 3:**
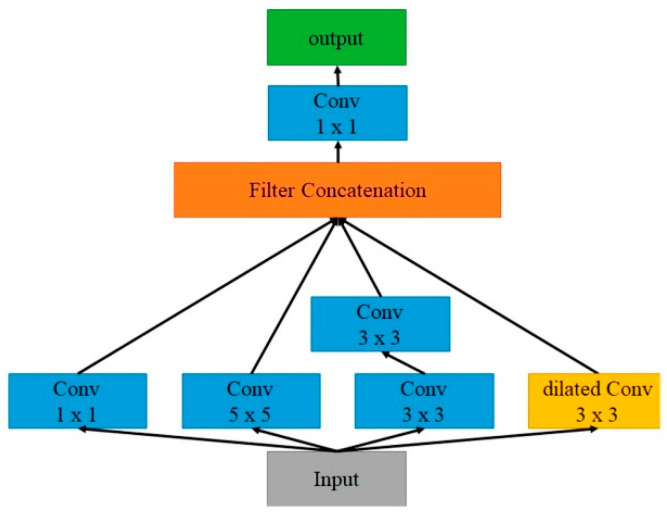
Dilated Inception block.

**Figure 4:**
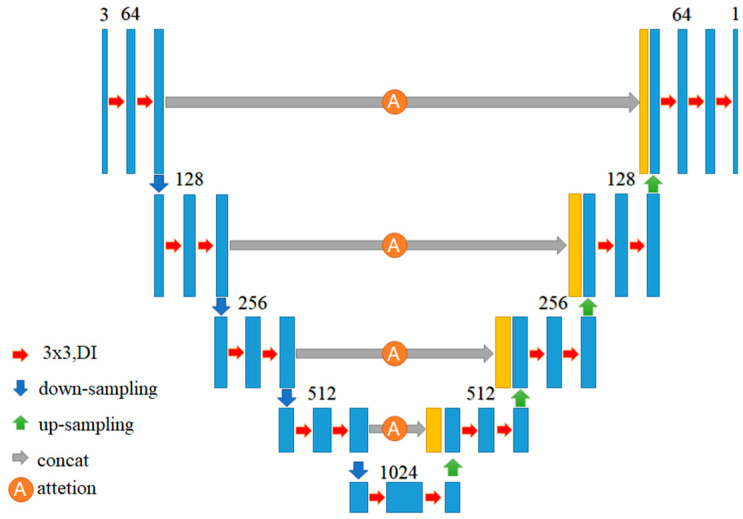
The architecture of the DIU-Net, where convolutional layers are replaced by DIs and the numbers indicate the number of channels.

In fact, the size of nuclei in pathological images is not uniform, so the segmentation network must be able to capture cross-scale features to focus on nuclei of different sizes. Experiments have confirmed that the Inception structure has the ability to capture features at different scales [29], but unfortunately, the pooling layer in the Inception structure weakens this ability of the overall model due to the information loss coming from pooling. To overcome this shortcoming, this study replaces the pooling layer of Inception with dilated convolution [30], and calls the replaced block, as shown in Figure 3, Dilated Inception (DI). To further strengthen the cross-scale description capability of the Attention U-Net, we replace the convolutional layers of the encoder and decoder of the Attention U-Net with the DI blocks. The adjusted architecture is called DIU-Net as shown in Figure 4.

To enhance the generalization ability of DIU-Net, it is necessary to avoid the operation of DIU-Net relying on the color information and relative brightness of the nucleus and the background which are less reliable. C3S was proposed to achieve this goal. By converting some training samples to complementary colors, DIU-Net learns to ignore the color and relative brightness of the nucleus and background and instead learns to use higher-level texture features for segmentation judgment. Pathological images are inherently characterized by the fact that the area of nuclei is much smaller than the background area, which leads to data imbalance. If an inappropriate loss function is used, it is easy for the trained segmentation network to pay more attention to the accuracy of non-nucleus regions, resulting in segmentation bias. Dice loss belongs to region-based loss and uses the relative overlap rate between the prediction result and the ground truth to quantify the loss. It is not affected by the size of the target object innately, so it is suitable for the loss of the segmentation network. However, using dice loss alone is prone to instability when the relative overlap rate between the prediction result and the ground truth is zero during the training process. To avoid this problem, this study added focal loss which belongs to distribution-based loss and can also help improve the problem of data imbalance. The dice loss is defined in Equation (1), where *N* is the total pixel number in one batch, ${ŷ}_{i}$ and $y_{i}$ denote the prediction result and the ground truth for pixel *i*, respectively. The ϵ term is used to ensure the loss function stability by avoiding the numerical issue of dividing by 0. The focal loss is defined in Equation (3).

(1) Ldice =1−∑i=1Nyi ŷi+ϵ∑i=1Nyi+ ŷ i+ϵ−∑i=1N(1−yi)(1− ŷi)+ϵ∑i=1N2−yi− ŷi+ϵ

(2) $${ỹ}_{i}=\left\{\begin{array}{c} {\;ŷ}_{i}\\ 1-{\;ŷ}_{i} \end{array}\begin{array}{c} if\;y_{i}=1\\ \;\;otherwise \end{array}\right.$$

(3) $$L_{\mathrm{focal}\;}=-\sum_{i=1}^{N}{\left(1-{ỹ}_{i}\right)}^{2}\log\left({ỹ}_{i}\right)$$

### 2.3. Classification Network

**Figure 5:**
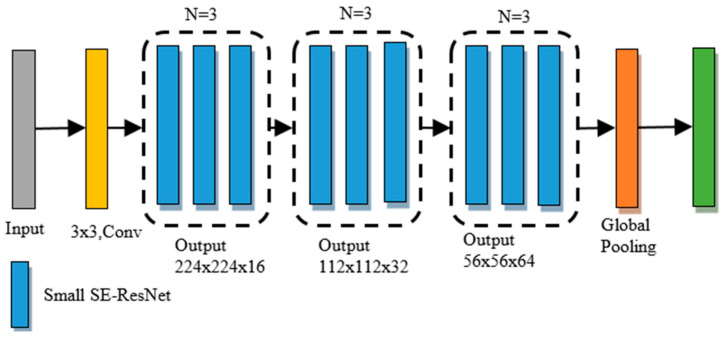
The architecture of the BHCNet-3.

In this study, BHCNet [22], which currently has outstanding breast tumor classification performance in the public dataset BreaKHis, was used as the classification network, and its architecture is shown in Figure 5. BHCNet-3 was used for benign and malignant tumor classification tasks. For the more difficult subtypes classification task, BHCNet-6 was used. The difference between the two networks is the network depth. Since the subtypes classification task is more difficult than the binary classification task, a deeper network is required to cope with it.

### 2.4. Training

The loss function of the overall network can be divided into two parts: segmentation loss $L_{seg}$ and classification loss $L_{c}$. The definition of segmentation loss $L_{seg}$ is defined in Equation (4) where $\lambda_{s}$ is the weight of focal loss. $L_{seg}$ shoulders our expectation for the output of the segmentation network, that is, to appropriately reflect the degree of attention to the nucleus in the image. The loss of benign/malignant binary classification is denoted as $L_{bce}$, which is defined in Equation (5), where *M* is the total image number in one batch for the binary classification task, ${ŷ}_{k}$ and $y_{k}$ denote the prediction result and the ground truth for image *k*, respectively. As for the loss of subcategory classification, it is denoted as $L_{mce}$, which is defined in Equation (6), where *K* is the total image number in one batch for a multi-class classification task, ${ŷ}_{m}$ represents the target class prediction result of image *m*. The total loss $L_{total}$ is defined in Equation (8) where $\lambda_{c}$ is the weight of classification loss $L_{c}$.

The training samples of the overall network in the training phase are partly from the segmentation training set and partly from the classification training set. The segmentation network and the classification network are jointly trained by the training samples of the two training sets. The training sample $I_{s}$ from the segmentation training set is fed into DIU-Net to obtain NFM. The corresponding $L_{\mathrm{seg}\;}$ is calculated through the ground truth of $I_{s}$, and then the weights of DIU-Net are corrected via $L_{\mathrm{seg}\;}$. The training sample $I_{c}$ from the classification training set is fed into DIU-Net to obtain NFM. After multiplying NFM and $I_{c}$ to obtain NFWI, then input to BHCNet to obtain classification prediction. The corresponding loss $L_{c}$ is calculated through the ground truth of $I_{c}$. Correcting the weights of BHCNet and DIU-Net via $L_{total}$:

(4) $$L_{\mathrm{seg}\;}=L_{\mathrm{dice}\;}+\lambda_{s}L_{\mathrm{focal}\;}$$

(5) $$L_{bce}=-\sum_{k=1}^{M}\left[y_{k}\log\left({\;ŷ}_{k}\right)+(1-y_{k}\right)\log\left(1-{\;ŷ}_{k}\right)]$$

(6) $$L_{mce}=-\sum_{m=1}^{K}\log\left({\;ŷ}_{m}\right)$$

(7) $$L_{c}=\left\{\begin{array}{c} L_{bce}\;,\;two\;classes\\ L_{mce},multi-class \end{array}\right.$$

(8) $$L_{total}=\left(1-\lambda_{c}\right)*L_{\mathrm{seg}\;}+\lambda_{c}*L_{c}$$

## 3. Experiments

In this study, accuracy (ACC) and dice coefficient were used as efficacy evaluation indicators. The segmentation experiment is divided into two parts: the training process with/without the C3S, in order to understand the benefits of this scheme. As for the verification method of the classification experiment, the same evaluation methods were adopted as other studies [20,22,24,31,32] using the BreaKHis dataset.

### 3.1. Datasets

Since few of the currently public breast cancer image datasets provide both the ground truth for nucleus segmentation and the ground truth for tumor types, the dataset used in this study is divided into a segmentation dataset and a classification dataset. The images of the segmentation dataset were collected from 4 sub-datasets, namely the UCSB dataset [33], the TNBC dataset [34], the 2018DSB dataset [35] and the MoNuSeg dataset [36]. There are 58 breast cancer images in the UCSB dataset, the image format is TIF, the resolution is 896 × 768, and the staining colors are relatively consistent. The TNBC dataset has 50 breast cancer images in PNG format with a resolution of 512 × 512, and their staining colors are relatively inconsistent. The 2018DSB dataset has 670 images of Spot nuclei, the image format is TIF, the resolution is 256 × 256 to 1388 × 1040 and the staining colors are also relatively inconsistent. The MoNuSeg dataset has 30 images. The tissue images are from patients with breast cancer, liver cancer, kidney cancer, prostate cancer, bladder cancer, colon cancer and gastric cancer. Since this dataset covers cells of different tissues, and the cell characteristics are highly variable, it is quite suitable for verifying the generalization ability of the segmentation network.

**Figure 6:**
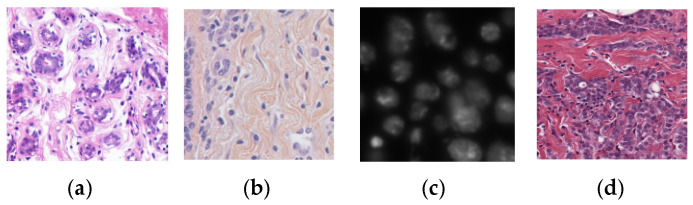
Sample images of four sub-datasets for segmentation: (**a**) UCSB; (**b**) TNBC; (**c**) 2018DSB; (**d**) MoNuSeg.

In terms of the appearance of staining, the images of the above four sub-datasets can be divided into two types: cells with a darker color than the background (referred to as the darker type) and cells with a lighter color than the background (referred to as the lighter type). Most of the images in the three sub-datasets UCSB, MoNuSeg and TNBC are of the darker type, while the 2018DSB sub-dataset is of the lighter type. Figure 6 shows sample images of each sub-dataset. In order to test the generalization ability of the segmentation network, this study made special arrangements for the training and testing datasets. UCSB and TNBC were used as segmentation training datasets, while MoNuSeg and 2018DSB were used as segmentation test datasets. A total of 80% of the data in the segmentation training dataset is used as training data, and the remaining 20% is used as validation data. Since the characteristics of the test data are quite different from the training data, if the test performance is good, it can be confirmed that the segmentation network model proposed in this study has excellent generalization ability.

The classification dataset is the BreaKHis dataset, which uses H&E staining, the image size is 700 × 400, and the image magnifications are 200× and 400×. Each image has a benign/malignant label and the corresponding subcategory label, for a total of 3833 images. The subcategories of benign tumors are adenosis (A), fibroadenoma (F), phyllodes tumor (PT) and tubular adenoma (TA). The malignant tumor subcategories are ductal carcinoma (DC), lobular carcinoma (LC), mucinous carcinoma (MC) and papillary carcinoma (PC). There are 2013 200× images, which are called the 200× dataset, and 1820 400× images, which are called the 400× dataset. Randomly select 70% from the 200× dataset as the 200× training set and the remaining 30% as the 200× test set. Likewise, randomly select 70% from the 400× dataset as the 400× training set and the remaining 30% as the 400× test set. The union of the 200× training set and the 400× training set is called the classification training set, and the union of the 200× test set and the 400× test set is called the classification test set.

### 3.2. Evaluating the Segmentation Performance

**Figure 7:**
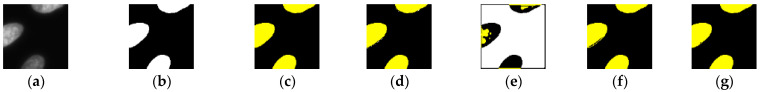
The segmentation results using different models without the C3S for a sample image of the 2018 DSB sub-dataset. The white pixel represents FP (false positive), while the yellow pixel represents FN (false negative). (**a**) Image; (**b**) Ground Truth; (**c**) U-Net; (**d**) Att-U-Net; (**e**) R2U-Net; (**f**) U-Net++; (**g**) Ours.

**Figure 8:**
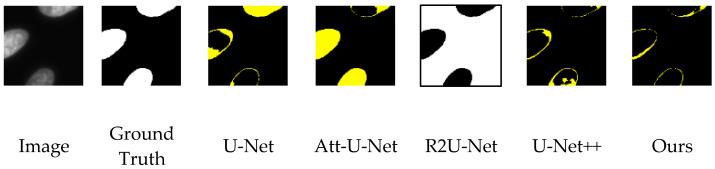
The segmentation results using different models with the C3S during the training phase for the same image in Figure 7.

U-Net [25] can be regarded as a representative model for medical image segmentation. In recent years, some improved architectures based on U-Net have been proposed, such as Attention U-Net (Att-U-Net) [26], R2U-Net [27] and U-Net++ [28]. These models all have quite good segmentation performance. This experiment compares the segmentation performance of the proposed DIU-Net with these models under the same training and testing conditions. Table 1 shows the test results of the training process of each model without using the C3S for training. The results show that our proposed model outperforms the other methods on both the validation set and the test set and R2U-Net has the worst segmentation effect. However, the performance of all models on the test set is not ideal. The main reason is that these models learn to rely on the color and relative brightness of the nucleus and background to make judgments during the training process, which reduces the performance of the test data with different characteristics. Figure 7 shows examples of the segmentation results of the 2018 DSB sub-dataset. It can be seen that all segmentation models cannot correctly segment the nucleus region. Table 2 shows the test results of the training process of each model using the C3S. It can be found that the segmentation performance of all models is greatly improved, indicating that the C3S can indeed improve the generalization ability of these models. The experimental results also show that our proposed model performs better than other methods in both the validation set and test set, and the segmentation effect of R2U-Net is still the worst. The actual segmentation performance can refer to the segmentation examples in Figure 8. Compared with Figure 7, the segmentation performance is indeed significantly improved.

To further understand the impact of different loss and C3S on the performance of the segmentation network, this part uses the performance of the validation set and the test set to conduct ablation experiments, and the results are shown in Table 3. It can be seen from the results that the performance of using only dice loss is worse than that of using focal loss, while the performance of combining dice loss with focal loss is the best. Furthermore, no matter what kind of loss is used, as long as the C3S is used in the training process, better segmentation performance can be obtained. The results show that the combination of dice loss and focal loss is beneficial to the optimization of the segmentation network. The C3S used in the training process can improve the generalization ability of the segmentation network.

### 3.3. Evaluating the Classification Performance

**Figure 9:**
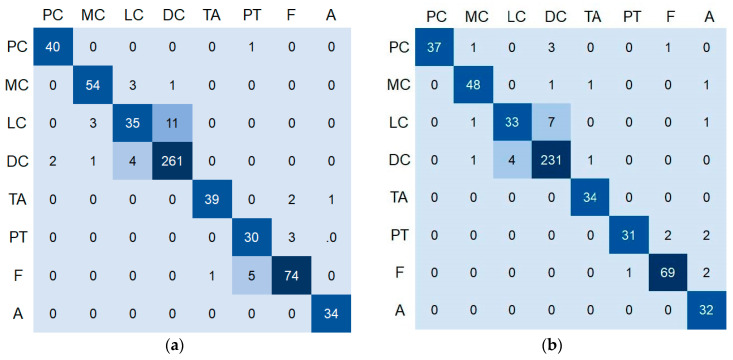
The multi-class confusion matrix of the proposed model for (**a**) 200× test set and (**b**) 400× test set.

Table 4 shows the results of the dichotomous classification of benign and malignant breast pathological images by various methods. CNN [20] means the binary/multi-class classification results using the CNN-based method of [20]. ResNet [21] means the binary/multi-class classification results using Inception and ResNet architectures proposed by [21]. BHCNet [22] is the baseline for classification performance comparison since our proposed method employs it as a classification module. NucDeep [24] means the binary/multi-class classification results using the method proposed by [24], in which a feature fusion approach with a support vector machine is used to classify breast tumor images. ResHist [31] is a residual learning-based CNN with 152 layers developed for breast tumor binary classification. myResNet-34 [32] is also a residual learning-based CNN derived from ResNet-34 via merging shallow features and using Leaky ReLU and Batch Normalization to enhance the malignancy-and-benign classification performance. The results show that the model proposed in this study has a higher classification accuracy than other methods whether in the 200× test set, 400× test set or the overall test set. The performance of various methods in the classification of benign and malignant subcategories of breast pathology images is shown in Table 5. The results show that the proposed model still has higher subcategory classification accuracy than other methods whether it is in the 200X test set, 400X test set or in the overall test set. This shows that the proposed strategy combining the degree of nucleus attention can indeed effectively improve classification performance. The multi-class confusion matrix of the proposed model is shown in Figure 9. From the confusion matrix, it can be found that LC is the most difficult category to be classified.

### 3.4. Ablation Study

In this ablation experiment, we want to understand the impact of several key factors on classification accuracy. The ablation experimental results are shown in Table 6. Omitting the C3S, the performance degradation is 0.56 and 1.42 for binary classification and multi-class classification, respectively. The results reveal that the improvement of segmentation performance by the C3S also indirectly boosts the classification performance. To understand the influence level of replacing the pooling layer of Inception with dilated convolution, the results with and without DI were compared. From Table 6, we can find that using DI the classification accuracy can be enhanced by 0.55 and 2.38 for binary and multi-class classification, respectively. This reflects that DI can indeed improve the cross-scale description ability of Attention U-Net for the nucleus in breast pathology images. To further understand the impact of the joint training of the segmentation network and the classification network on the accuracy of the binary classification and multi-class classification, a control version, the separated training version, was designed to compare the classification performance of the two. This version trains the segmentation network and the classification network separately and then concatenates them after the training is complete. The comparison results are shown in Table 6. The classification performance of the joint training version outperforms the separated training versions by 6.32 and 18.11 on the binary and multi-class tasks, respectively. This substantial performance improvement is due to two factors. One is that the soft segmentation results of the segmentation network allow the classification network to focus more on the nucleus regions. The other is that the joint training scheme gives classification results, which can not only be fed back to optimize the classification network, but also optimize the segmentation network to generate the corresponding NFM that is more conducive to classification performance.

**Figure 10:**
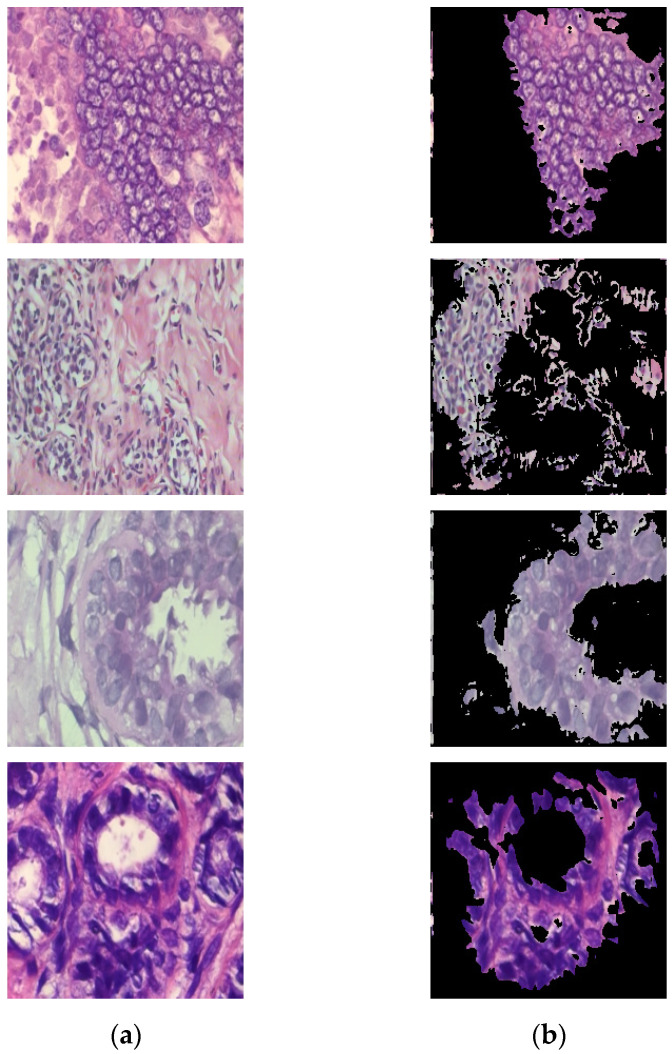
The segmentation results of the proposed method: (**a**) the input images; (**b**) segmented results of our method.

**Figure 11:**
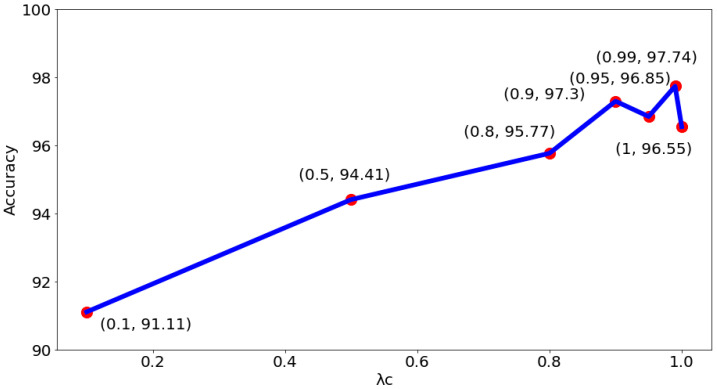
The impact of choosing different $\lambda_{c}$ on accuracy for binary classification.

**Figure 12:**
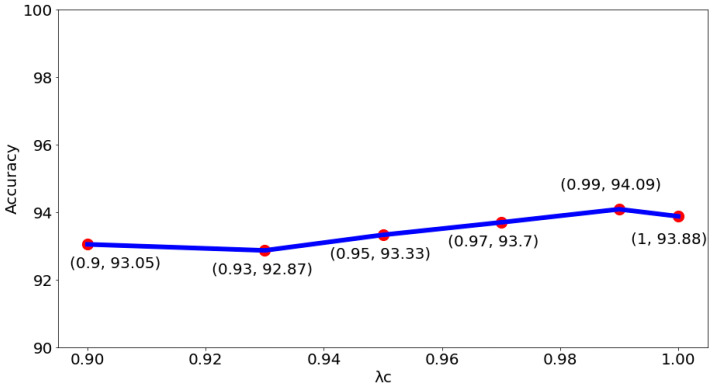
The impact of choosing different $\lambda_{c}$ on accuracy for multi-class classification.

In addition to yielding superior classification results, the proposed method has the side benefit of being able to provide model visualization. That is, the areas concerned by the model can be displayed to the pathologist for reference (as shown in Figure 10), thereby enhancing the physician’s confidence in the classification results. It can also be found from Figure 10 that the segmentation results of the proposed method cover almost all the nucleus regions. This also shows the effectiveness of the proposed method for nucleus segmentation. To understand the impact of choosing different $\lambda_{c}$ on the system performance, tests for different $\lambda_{c}$ were proceeded. Figure 11 shows the test results of the binary classification task. The results show that when $\lambda_{c}$ is set to 0.99, there is the best classification performance. As for the test results of the subcategory classification task, as shown in Figure 12, the results also show that the best classification performance is obtained when $\lambda_{c}$ is set to 0.99. This means that the hyperparameter $\lambda_{c}$ of the proposed model is quite stable, whether facing binary or subcategory tasks. This stable property indirectly reflects the value of the proposed model in clinical applications.

## 4. Conclusions

Inspired by pathologists’ interpretation of pathological images, this study proposed an automatic classification model of breast pathological images that combines a segmentation network and classification network. The soft segmentation results are generated by the segmentation network to simulate the pathologist’s relative attention to the viewing mode of the nucleus regions, and then the weighted images are input into the classification network for classification. Considering the phenomenon of different sizes of nuclei in pathological images, the DIU-Net proposed in this study has excellent cross-scale description ability, so that nuclei of different sizes can correspond to higher attention coefficients. In addition, to make the segmentation network have better generalization ability, C3S was used in the training phase to guide the segmentation network to avoid learning low-level features such as color or relative brightness and instead learn higher-level texture features to identify nuclei. With the background, the generalization ability of the segmentation network has been successfully improved. Furthermore, dice loss and focal loss were used to successfully overcome the data imbalance caused by the disparate area ratio of nuclei to the background. This study utilized a joint training scheme so that the output of the classification network can not only be used to optimize the classification network itself, but also optimize the segmentation network to further strengthen the classification performance of the model. The experimental results show that the model proposed in this study outperforms the existing classification models for both 200× and 400× pathological images in both binary and subcategory classification tasks. In addition, the proposed model has the side benefit of being able to provide the areas concerned by the model. This visualization can enhance the pathologist’s confidence in the model’s classification results. The greatest contribution of the model developed in this study is that it can provide pathologists with excellent classification results and at the same time provide information on the area of interest of the model, thereby assisting pathologists in making decisions. The main drawback of this model is that it cannot be applied to small-magnification pathological images because this model must first softly segment the nucleus area, and small-magnification pathological images cannot clearly reveal the nucleus.

## Figures and Tables

**Table 1:** The test results of different segmentation models without C3S in the training phase.

		U-Net	Att-U-Net	R2U-Net	U-Net++	Ours
	
Validation Set						
Dice Coefficient		0.69	0.68	0.35	0.68	**0.70**
Test Set						
Dice Coefficient		0.39	0.40	0.36	0.36	**0.41**

**Table 2:** The test results of different segmentation models with C3S in the training phase.

		U-Net	Att-U-Net	R2U-Net	U-Net++	Ours
	
Validation Set						
Dice Coefficient		0.84	0.84	0.24	0.85	**0.85**
Test Set						
Dice Coefficient		0.50	0.53	0.33	0.52	**0.54**

**Table 3:** Ablation experiments on the segmentation network.

Focal Loss	Dice Loss	Dice Coefficient (without C3S)	Dice Coefficient (with C3S)
Validation Set			
✓	✗	0.66	0.84
✗	✓	0.66	0.82
✓	✓	**0.70**	**0.85**
Test Set			
✓	✗	0.41	0.47
✗	✓	0.34	0.44
✓	✓	**0.41**	**0.54**

**Table 4:** Binary classification accuracy comparison.

Methods	200×	400×	Total
CNN [20]	96.36	95.97	96.16
ResNet [21]	93.64	93.16	93.4
BHCNet [22]	97.2	96.96	97.04
NucDeep [24]	96.21	✗	96.21
ResHist [31]	91.15	86.27	✗
myResNet-34 [32]	90.47	88.79	✗
**Ours**	**97.24**	**98.19**	**97.74**

**Table 5:** Multi-class classification accuracy comparison.

Methods	200×	400×	Total
CNN [20]	80.83	81.03	80.93
ResNet [21]	76.54	79.58	78.06
BHCNet [22]	92.27	91.15	91.71
NucDeep [24]	63.3	✗	63.3
**Ours**	**93.75**	**94.43**	**94.09**

**Table 6:** Ablation experiment on classification accuracy.

Methods	Binary Classification	Multi-Class Classification
**Without C3S**	97.18	92.67
**Without DI**	97.04	91.71
**Separated training version**	91.42	75.98
**Ours**	**97.74**	**94.09**
